# One-year postprocedural quality of life following mitral valve surgery: data from The Netherlands Heart Registration

**DOI:** 10.1093/icvts/ivae051

**Published:** 2024-03-23

**Authors:** Samuel Heuts, Jules R Olsthoorn, Saskia Houterman, Maaike M Roefs, Jos G Maessen, Peyman Sardari Nia, Cardiothoracic Surgery Registration Committee of the Netherlands Heart Registration, Bramer, van Boven, Vonk, Koene, Bekkers, Hoohenkerk, Markou, de Weger, Segers, Porta, Speekenbrink, Stooker, Li, Daeter, van der Kaaij, Douglas

**Affiliations:** Department of Cardiothoracic Surgery, Maastricht University Medical Center+, Maastricht, Netherlands; Cardiovascular Research Institute Maastricht (CARIM), Maastricht University, Maastricht, Netherlands; Department of Cardiothoracic Surgery, Maastricht University Medical Center+, Maastricht, Netherlands; Cardiovascular Research Institute Maastricht (CARIM), Maastricht University, Maastricht, Netherlands; Department of Cardiothoracic Surgery, Catharina Hospital, Eindhoven, Netherlands; Netherlands Heart Registration, Utrecht, Netherlands; Netherlands Heart Registration, Utrecht, Netherlands; Department of Cardiothoracic Surgery, Maastricht University Medical Center+, Maastricht, Netherlands; Cardiovascular Research Institute Maastricht (CARIM), Maastricht University, Maastricht, Netherlands; Netherlands Heart Registration, Utrecht, Netherlands; Department of Cardiothoracic Surgery, Maastricht University Medical Center+, Maastricht, Netherlands; Cardiovascular Research Institute Maastricht (CARIM), Maastricht University, Maastricht, Netherlands

**Keywords:** Mitral valve surgery, Quality of life, Sternotomy, Minimally invasive mitral valve surgery

## Abstract

**OBJECTIVES:**

The aim of surgical treatment of mitral valve disease is to reverse heart failure and to restore life expectancy and quality of life (QoL). In mitral valve surgery, QoL has not been studied extensively, especially regarding the surgical approach. The current study aimed to evaluate QoL after mitral valve surgery through full sternotomy and a minimally invasive approach (minimally invasive mitral valve surgery).

**METHODS:**

All patients undergoing mitral valve surgery between 2013 and 2018 through sternotomy or a minimally invasive mitral valve surgery approach (right anterolateral mini-thoracotomy, sternal-sparing), with or without concomitant tricuspid valve surgery, surgical ablation or atrial septal defect closure were eligible for inclusion in this multicentre nationwide registry in the Netherlands. QoL was measured using the 12- and 36-item short form surveys, before surgery and postoperatively at 1 year. Independent predictors for loss of QoL were evaluated.

**RESULTS:**

A total of 485 patients were included (full sternotomy: *n* = 276, and minimally invasive mitral valve surgery: *n* = 209). Overall, patients experienced a significant increase in physical component score [56 (42–75) vs 74 (57–88), *P* < 0.001] and mental component score at 1 year [63 (52–74) vs 70 (59–86), *P* < 0.001]. Baseline QoL scores and new onset of atrial arrhythmia were independently associated with a clinically relevant reduction in physical and mental QoL.

**CONCLUSIONS:**

Mitral valve surgery is associated with significant improvement in physical and mental QoL. Baseline QoL scores and new onset of atrial arrhythmia are associated with a clinically relevant reduction in postoperative QoL.

**Figure:**
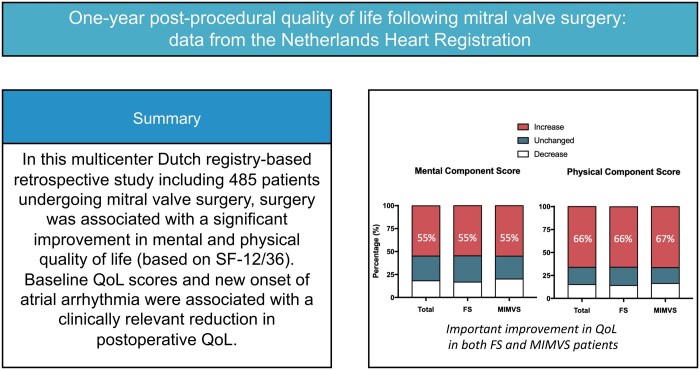

## INTRODUCTION

Severe mitral valve (MV) disease, comprising both mitral stenosis and mitral regurgitation, is associated with a reduced life expectancy, significant morbidity and development of symptoms affecting daily life [1]. Consequently, the aim of surgical treatment of MV disease is to restore life expectancy, reverse the development of heart failure, resolve symptoms and improve quality of life (QoL). QoL has been evaluated previously in the surgical treatment of MV disease. These studies mainly focused on differences in QoL between mitral valve repair and replacement, transcatheter procedures and conservative treatment [2], and biological and mechanical prostheses [3]. Recently, a randomized controlled trial evaluated QoL after a minimally invasive approach as compared to sternotomy, and did not observe a statistically significant difference in QoL after 12 weeks [4].

Indeed, patient-reported outcomes measures (PROM) play an increasingly important role in decision-making, especially in the light of the ageing population, and the advent of transcatheter and minimally invasive techniques. For surgical procedures on the MV, minimally invasive MV surgery (MIMVS) has proven to be a valuable alternative to a full sternotomy (FS) approach, with comparable short-term outcomes [5], and equivalent long-term survival [6, 7]. By circumventing FS and its associated drawbacks, MIMVS has the potential advantage—especially in the re-operative setting [8]—of a reduced complication rate, an enhanced recovery and, potentially, an improved QoL. Of note, MV-associated procedures, such as tricuspid valve surgery, surgical ablation for atrial fibrillation (AF) and closure of an atrial septal defect, can also be performed through a minimally invasive approach [9].

In the current study, we aim to evaluate QoL following MV surgery in patients undergoing FS or MIMVS using a nationwide registry.

## MATERIALS AND METHODS

### Source of study data and ethical statement

The Netherlands Heart Registration (NHR) was described in detail before [10]. In short, reporting of interventions to the NHR is compulsory, using a variety of mandatory and non-mandatory parameters, including patient characteristics, intervention variables and outcomes. As these registry data are fully anonymized, institutional review board approval was not applicable. This study is in line with all institutions’ ethical policies and standards.

### Inclusion

Although most parameters are mandatory to report, QoL assessment was not compulsory. Consequently, not all patients in the current registry completed both pre- and postoperative QoL assessment between 2013 and 2018.

In the current study, all adult patients undergoing elective, primary MV surgery, with or without MV-related procedures (i.e. tricuspid valve surgery, surgical ablation for AF or closure of an atrial septal defect), with (i) a registered approach (i.e. FS or MIMVS) and (ii) pre- and postoperative QoL assessment (>50% completed) between 2013 and 2018 were included. Inherently, this implies only one-year survivors were included. Of note, MIMVS was defined as a surgical procedure on the MV, performed through a sternal-sparing approach, through a right anterolateral mini-thoracotomy.

Between 2013 and 2018, 5394 patients underwent MV surgery in the Netherlands, of which 2914 had a registered approach (FS or MIMVS). Patients undergoing reoperative surgery were excluded from the current analysis (*n* = 413). Of the 2501 patients undergoing primary surgery with a registered surgical approach, 485 patients completed both pre- and postoperative QoL assessment (exclusion of 2016 non-responders, flowchart in Supplementary Material S2).

### Study design

The current study is a retrospective multicentre cohort study of prospectively collected registry data from 11 Dutch cardiothoracic centres, included between 2013 and 2018.

### Quality of life assessment

In the NHR database, both the 12-item and 36-item short form survey (SF-12 and SF-36, respectively) are included, for which the specific questions can be found elsewhere [11, 12]. The SF-36 was developed in the early 1990s, objectively assessing health-related QoL [12]. In the SF-36, QoL is divided in a physical component score (PCS) and mental component score (MCS). For PCS and MCS, these scores are assessed in subdomains (physical functioning, role physical, bodily pain and general health, for PCS, and social functioning, role emotional, vitality and mental health, for MCS).

In a later era, the SF-36 was reduced to SF-12 incorporating 12 identical questions of the SF-36, assessing the same specific subdomains. Several studies have confirmed that outcomes of both surveys were virtually identical, for cardiac and non-cardiac patients [11]. As such, both surveys can be used interchangeably.

In the NHR database, the SF-36 was used by 10 centres, while 1 centre reported QoL based on the SF-12. Furthermore, QoL was assessed directly before surgery, and at 1-year after surgery (between 10 and 14 months postoperatively).

### Outcomes

Primary outcomes were pre- and postoperative (1-year) QoL (PCS and MCS) in absolute points for both component scores, and differences in stratified QoL changes between both approaches. Although definitions of stratified changes in SF scores should be based on standardized mean differences in the dataset [13], a large study of randomized trials defined corresponding raw SF score cut-offs of 0–4 points for a small effect, 4–10 points for a moderate effect and >10 points for a large effect [13]. For the current study, the minimal clinically important difference was defined as ≥5 points difference. Consequently, changes in QoL were stratified in reduced QoL (≥5 points decrease), similar QoL (<5 points difference) or increased QoL (≥5 points QoL).

The secondary aim was to evaluate predictors of a reduction of QoL (≥5 points decrease in PCS or MCS).

The clinical postprocedural outcomes of the entire Dutch MV surgical cohort were reported extensively before, and can be found elsewhere [6]. However, to comprehend the outcomes of the current cohort, which only included patients with completed pre- and postoperative QoL questionnaire forms, the clinical outcomes of this subgroup were evaluated and presented as well.

### Statistical analysis

Continuous data were presented as means and standard deviations or medians with 25th and 75th percentile. We prespecified to test the distribution of the data by visual assessment of P–P plots and by the Kolmogorov–Smirnov’s test (for which *P* < 0.05 indicated a statistically significant deviation of the normal distribution). Student’s *T*-test (in case of a normal distribution) or Mann–Whitney *U*-test (when data were not distributed normally) was used for unrelated comparisons, while the paired *T*-test (normal distribution) or Wilcoxon signed rank test (non-normal distribution) were used for paired samples. Categorical data were presented as absolute numbers and percentages (%) and compared using the χ^2^-test (unpaired, in case of *n* < 5 Fisher’s exact test) or McNemar test (for paired samples). Sensitivity analyses were performed for patients undergoing mitral valve repair (as repair itself is associated with improved QoL [14]), and truly isolated MV surgery. Furthermore, responders and non-responders were compared to evaluate potential selection bias. Of note, we did not perform statistical tests to assess the difference between FS and MIMVS in unadjusted analyses, as the 2 treatment groups may be subjected to selection bias, allocation bias and unmeasured confounding. As such, no *P*-values are reported for these outcomes.

A univariable logistic regression model was applied based on the available data, incorporating baseline and procedural characteristics, major postoperative complications (defined as stroke, myocardial infarction, renal failure or vascular complications), re-exploration for bleeding and length of hospital stay, amongst others. A clinically relevant reduction of QoL (defined as >5 points decrease) was used as the dependent variable. When *P* < 0.20 in univariable analysis, this covariate was included in the multivariable model. However, regardless of the *P*-value in the univariable model, surgical approach (i.e, MIMVS as a parameter) was always forced into the multivariable model. Results of the logistic regression models were presented as odds ratio (OR) and corresponding 95% confidence intervals (CI). Model discrimination was tested in a receiver operating characteristic analysis, of which an area under the curve <0.7 indicated poor discrimination, 0.7–0.8 acceptable discrimination and >0.8 excellent discrimination. Hosmer and Lemeshow tests were performed to evaluate goodness-of-fit.

All statistical analyses were performed using SPSS software (V27, IBM, Armonk, NY, USA).

## RESULTS

### Baseline characteristics

In total, 485 patients, who completed both pre- and postoperative QoL assessment, were included. A comparison between responders (*n* = 485) and non-responders (*n* = 2016) is presented in Supplementary Material S3. The response rate was 19.4% (485 responders of 2501 eligible patients). In general, non-responders had higher operative risk (as defined by logistic EuroSCORE), primarily driven by an increased rate of concomitant tricuspid valve surgery, the presence of more females, and a lower left ventricular ejection fraction.

Responders were divided into FS group (*n* = 276, 56.9%) and MIMVS group (*n* = 209, 43.1%). Baseline characteristics are presented in Table 1. Patients undergoing FS showed a tendency towards a higher estimated surgical risk, based on a logistic EuroSCORE [3.24 (2.08–5.61) vs 2.76 (1.94–5.10), *P* = 0.05].

**Table 1:** Baseline and procedural characteristics of all included patients, subdivided into FS and MIMVS groups

	Total (*n* = 485)	FS (*n* = 276)	MIMVS (*n* = 209)	*P*-value
Baseline				
Age (years)	66.0 (58.0–72.0)	66.0 (58.0–72.0)	65.0 (58.0–72.0)	0.48
Female sex (%)	190 (39.2%)	102 (37.0%)	88 (42.1%)	0.25
BMI (kg/m^2^)	25.7 (23.2–28.0)	25.6 (23.1–27.8)	25.7 (23.5–28.4)	0.23
Diabetes mellitus (%)	20 (4.1)	14 (5.1)	6 (2.9)	0.23
LVEF >50% (%)	395 (81.4)	218 (79.0)	177 (84.7)	0.11
COPD (%)	35 (7.2)	27 (9.8)	8 (3.8)	**0.01**
Peripheral arterial disease (%)	8 (1.6)	6 (2.2)	2 (1.0)	0.48
Endocarditis (%)	3 (0.6)	3 (1.1)	0 (0.0)	0.26
Recent myocardial infarction (<90 day, %)	2 (0.4)	2 (0.7)	0 (0)	0.51
Estimated glomerular filtration rate (ml/min)	76 (18)	74 (17)	77 (19)	0.07
Severe renal dysfunction, *n* (%)	2 (0.4)	1 (0.4)	1 (0.5)	0.99
Pulmonary hypertension), *n* (%)	39 (8.0%)	27.0 (9.8)	12 (5.7)	0.11
Procedure				
MVr (%)	391 (80.6)	226 (81.9)	165 (78.9)	0.42
MVR (%)	94 (19.4)	50 (18.1)	44 (21.1)	0.42
Rhythm surgery (%)	97 (20.0)	76 (27.5)	21 (10.0)	**<0.001**
ASD closure (%)	15 (3.1)	11 (4.0)	4 (1.9)	0.19
TV surgery (%)	57 (11.8)	50 (18.1)	7 (3.3)	**<0.001**
Mortality risk				
EuroSCORE (log)	3.07 (2.08–5.38)	3.24 (2.08–5.61)	2.76 (1.94–5.10)	0.05

Continuous variables are presented as median and (25–75th percentile) or as mean (SD).

ASD: atrial septal defect; BMI: body mass index; COPD: chronic obstructive pulmonary disease; EuroSCORE: European system for cardiac operative risk evaluation; FS: full sternotomy; LVEF: left ventricular ejection fraction; MIMVS: minimally invasive mitral valve surgery; MVr: mitral valve repair; MVR: mitral valve replacement; SD: standard deviation; TV: tricuspid valve.

Bold denotes statistical significance.

### Pre- and postoperative quality of life

In the overall cohort, patients undergoing MV surgery (*n* = 485) experienced a significant increase in the PCS [56 (42–75) vs 74 (57–88), *P* < 0.001] and MCS at 1 year [63 (52–74) vs 70 (59–86), *P* < 0.001]. Table 2 presents the overall PCSs and MCSs, with a subdivision per specific domain.

**Table 2:** Pre- and postoperative quality of life of mitral valve patients, based on SF-12 and SF-36 questionnaires

	Preoperative	Postoperative	*P*-value
	Total (*n* = 485)		
PCS	56 (42–75)	74 (57–88)	<0.001
Subdomains			
Physical functioning	30 (50–80)	85 (50–100)	
Role physical	38 (19–69)	63 (38–88)	
Bodily pain	90 (67–100)	100 (75–100)	
General health	50 (45–70)	60 (50–80)	
MCS	63 (52–74)	70 (59–86)	<0.001
Subdomains			
Vitality	50 (31–75)	63 (50–75)	
Social functioning	75 (50–100)	88 (75–100)	
Role emotional	67 (25–100)	75 (50–100)	
Mental health	65 (50–85)	75 (55–90)	
	FS (*n* = 276)		
PCS	57 (43–73)	73 (57–88)	<0.001
Subdomains			
Physical functioning	60 (39–85)	85 (60–95)	
Role physical	25 (9–56)	50 (25–88)	
Bodily pain	89 (59–100)	100 (78–100)	
General health	60 (50–75)	68 (50–85)	
MCS	66 (52–78)	73 (62–89)	<0.001
Subdomains			
Vitality	56 (38–75)	63 (50–75)	
Social functioning	75 (50–100)	88 (75–100)	
Role emotional	50 (25–92)	75 (25–100)	
Mental health	80 (65–90)	85 (70–93)	
	MIMVS (*n* = 209)		
PCS	56 (38–75)	75 (57–88)	<0.001
Subdomains			
Physical functioning	50 (25–75)	75 (50–100)	
Role physical	50 (25–75)	75 (50–100)	
Bodily pain	100 (75–100)	100 (75–100)	
General health	50 (25–50)	50 (50–75)	
MCS	63 (51–95)	69 (56–81)	<0.001
Subdomains			
Vitality	50 (25–75)	50 (25–75)	
Social functioning	75 (50–100)	75 (50–100)	
Role emotional	75 (50–100)	88 (63–100)	
Mental health	50 (38–63)	63 (50–75)	

FS: full sternotomy; MCS: mental component score; MIMVS: minimally invasive mitral valve surgery; PCS: physical component score; SF-12: 12-item short form survey; SF-36: 36-item short form survey.

Both FS and MIMVS patients experienced an increase in physical QoL [57 (43–73) vs 73 (57–88) and 56 (38–75) vs 75 (57–88), both *P* < 0.001]. For mental QoL, similar results were observed [66 (52–78) vs 73 (62–89) and 63 (51–69) vs 69 (56–81), both *P* < 0.001].

### Differences in quality of life change following sternotomy and minimally invasive mitral valve surgery

The absolute changes (delta’s) between baseline and 1-year QoL for FS and MIMVS were 11 points (interquartile range 2–23) and 13 points for physical QoL, and 7 points (–2 to 19) and 6 (–3 to 19) for mental QoL, respectively.

**Figure 1:**
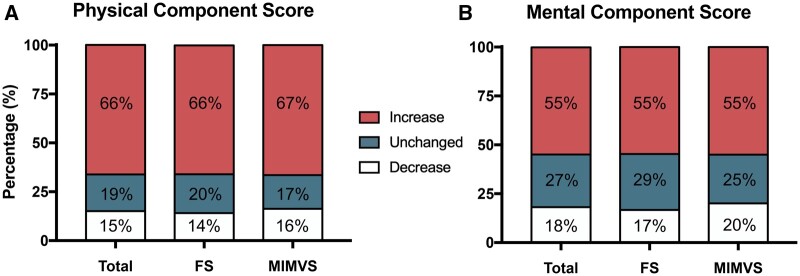
Clinically important changes in quality of life for physical component score (**A**) and mental component score (**B**), for the overall cohort, and stratified for patients undergoing FS and MIMVS. FS: full sternotomy; MIMVS: minimally invasive mitral valve surgery.

Clinically important changes in QoL can be appreciated in Fig. 1, with an important increase in QoL for both approaches.

### Sensitivity analyses

Subgroup and sensitivity analysis were performed for mitral valve repair patients [total *n* = 391 (80.6%), FS *n* = 226 (57.8%), MIMVS *n* = 165 (42.2), Supplementary Materials S4 and S5], and truly isolated MV surgery [total *n* = 353 (72.8%), FS *n* = 169 (47.9%), MIMVS *n* = 184 (52.1%), Supplementary Materials S6 and S7]. These findings confirmed the robustness of the previously observed data.

### Predictors of loss of quality of life

Potentially important baseline, operative and clinical outcomes related to QoL assessment were used for regression analyses. A uni- and multivariable logistic regression model was realized for physical QoL (Table 3), resulting in the identification of a significant association between decrease of QoL and baseline PCS per point increase (OR 1.05 95% CI 1.03–1.06), and new-onset arrhythmia (OR 2.58, 95% CI 1.42–4.72). MIMVS or FS as an approach was not associated with a decrease in physical QoL. Receiver operating characteristic analysis confirmed good discrimination of the model (area under the curve (AUC) = 0.75), and Hosmer and Lemeshow test confirmed goodness-of-fit (*P* = 0.66).

**Table 3:** Uni- and multivariable logistic regression analysis for a decrease in PCS

	Univariable		Multivariable
Covariates	OR (95% CI)	*P*-value	OR (95% CI)
MIMVS	1.18 (0.72–1.95)	0.52	1.54 (0.86–2.75)
Female gender	0.68 (0.40–1.15)	0.15	1.07 (0.59–1.91)
Age	0.99 (0.97–1.01)	0.40	
LVEF <50%	0.58 (0.27–1.20)	0.14	0.80 (0.36–1.76)
Diabetes mellitus	0.99 (0.28–3.49)	0.99	
COPD	0.94 (0.35–2.50)	0.90	
PHT	1.30 (0.53–2.97)	0.60	
Peripheral arterial disease	0.80 (0.10–6.63)	0.84	
MVr	1.26 (0.65–2.46)	0.49	
Rhythm surgery	0.67 (0.34–1.33)	0.26	
ASD closure	1.43 (0.39–5.19)	0.59	
TV surgery	1.07 (0.50–2.28)	0.87	
Baseline PCS	1.05 (1.03–1.06)	**<0.001**	1.05 (1.03–1.06)
LOHS	1.01 (0.95–1.07)	0.86	
Major complications ^a^	1.58 (0.66–3.78)	0.31	
New onset arrhythmia	1.71 (1.03–2.87)	**0.04**	2.58 (1.42–4.72)
Re-exploration for bleeding	1.02 (0.99–1.05)	0.27	

a Major complications defined as stroke, myocardial infarction, renal failure or vascular complications. Bold denotes statistical significance.

ASD: atrial septal defect; CI: confidence interval; COPD: chronic obstructive pulmonary disease; LOHS: length of hospital stay; LVEF: left ventricular ejection fraction; MIMVS: minimally invasive mitral valve surgery; MVr: mitral valve repair; OR: odds ratio; PCS: physical component score; PHT: pulmonary hypertension, TV: tricuspid valve.

A similar model was realized for mental QoL (Table 4), identifying baseline MCS per point increase (OR 1.03, 95% CI 1.02–1.05) to be associated with a decrease in mental QoL at 1 year. MIMVS or FS as an approach was not associated with a decrease in mental QoL. For this model, discrimination was poor, as demonstrated by an AUC of 0.65 in receiver operating characteristic analysis, but Hosmer and Lemeshow test confirmed goodness-of-fit (*P* = 0.96).

**Table 4:** Uni- and multivariable regression analysis for a decrease in MCS

	Univariable		Multivariable
Covariates	OR (95% CI)	*P*-value	OR (95% CI)
MIMVS	1.26 (0.79–1.99)	0.33	1.45 (0.89–2.36)
Female gender	1.16 (0.72–1.85)	0.54	
Age	0.99 (0.97–1.01)	0.31	
LVEF <50%	1.16 (0.65–2.07)	0.61	
Diabetes mellitus	1.53 (0.54–4.33)	0.42	
COPD	0.93 (0.38–2.31)	0.87	
PHT	1.18 (0.52–2.67)	0.69	
Peripheral arterial disease	0.64 (0.08–5.27)	0.68	
MVr	1.21 (0.66–2.22)	0.54	
Rhythm surgery	0.72 (0.39–1.33)	0.30	
ASD closure	0.31 (0.41–2.42)	0.27	
TV surgery	0.96 (0.46–1.97)	0.90	
Baseline MCS	1.03 (1.02–1.05)	**<0.001**	1.03 (1.02–1.05)
LOHS	1.00 (0.95–1.06)	0.91	
Major complications	1.23 (0.52–2.94)	0.64	
New onset arrhythmia	1.06 (0.65–1.75)	0.81	
Re-exploration for bleeding	0.98 (0.90–1.06)	0.55	

a Major complications defined as stroke, myocardial infarction, renal failure or vascular complications. Bold denotes statistical significance

ASD: atrial septal defect; CI: confidence interval; COPD: chronic obstructive pulmonary disease; LOHS: length of hospital stay; LVEF: left ventricular ejection fraction; MIMVS: minimally invasive mitral valve surgery; MCS: mental component score; MVr: mitral valve repair; OR: odds ratio; PHT: pulmonary hypertension, TV: tricuspid valve.

Of note, major complications (defined as stroke, myocardial infarction, renal failure or vascular complications) were not statistically significantly associated with a decreased QoL.

### Clinical outcomes

Post-procedural outcomes (at 30 days) were presented in Table 5, with an important difference in new-onset arrhythmia, a complication which was also significantly associated with a decrease in physical QoL (Table 3).

**Table 5:** Postprocedural clinical outcomes at 30 days

	Total (*n* = 485)	FS (*n* = 276)	MIMVS (*n* = 209)	*P*-value
Pneumonia, *n* (%)	12 (2.5)	8 (2.9)	4 (1.9)	0.49
Urinary tract infection, *n* (%)	4 (0.8)	3 (1.1)	1 (0.5)	0.64
Reintubation due to respiratory insufficiency, *n* (%)	1 (0.2)	0 (0)	1 (0.5)	0.43
Prolonged intubation (>24 h), *n* (%)	7 (1.4)	5 (1.8)	2 (1.0)	0.70
Re-admission to ICU, *n* (%)	4 (0.8)	2 (0.7)	2 (1.0)	0.99
All stroke, *n* (%)	8 (1.6)	7 (2.5)	1 (0.5)	0.15
Stroke with neurological deficit, *n* (%)	6 (1.2)	5 (1.8)	1 (0.5)	0.24
Stroke without neurological deficit, *n* (%)	2 (0.4)	2 (0.7)	0 (0)	0.51
Kidney failure, *n* (%)	4 (0.8)	4 (1.4)	0 (0)	0.14
Gastro-intestinal complications, *n* (%)	3 (0.6)	2 (0.7)	1 (0.5)	0.99
Vascular complications, *n* (%)	2 (0.4)	2 (0.7)	0 (0)	0.51
New-onset arrhythmia, *n* (%)	149 (30.7)	123 (44.6)	26 (12.4)	<0.001
Re-exploration for bleeding (within 30 days), *n* (%)	19 (3.9)	8 (2.9)	11 (5.3)	0.18
Deep sternal wound infection, *n* (%)	1 (0.2)	1 (0.4)	0 (0)	0.99

FS: full sternotomy; ICU: intensive care unit; MIMVS: minimally invasive mitral valve surgery.

## DISCUSSION

The current study comprehensively evaluated QoL after mitral valve surgery in general, and by surgical approach in particular. Overall, patients improve significantly in terms of physical and mental QoL 1 year after surgery, and we found baseline QoL scores and new onset of atrial arrhythmia to be independently associated with a reduction in both physical and mental QoL.

Our findings are in line with the recent randomized controlled trial performed by Akowuah *et al*. the UK Mini trial [4]. In this randomized controlled trial, MIMVS was compared to FS for patients undergoing MV surgery, and the procedures were performed by experienced surgeons, resulting in few complications and an excellent repair rate. Although a statistically significant difference in QoL as measured by the SF-36 was observed after 6 weeks, this difference disappeared at the time of the primary end-point’s analysis at 12 weeks. In line with our findings, both MIMVS and FS provided sustainable improvement in QoL in the long term. Based on the findings from the UK Mini trial, there may be benefit of MIMVS regarding QoL in the very short term, but this is attenuated over a longer period and the surgical approach does not seem to influence longer-term QoL.

As the patient’s perspective can be regarded as a subjective opinion, it is imperative to measure these PROMs using the most objective modalities. For the assessment of QoL, the SF-36—and later on SF-12—was originally developed, incorporating not only health perception using physical measures, but also metrics for social, emotional and mental wellbeing [15]. Although physical and mental health are inseparably related, their outcome scores are reported separately using the SF scales. In the current study, overall, patients improved significantly in terms of physical QoL. This applied to patients undergoing both MIMVS and FS approaches. Although this improvement was a result of an increase in physical QoL over every physical subdomain, it was most notable in patients’ ‘physical functioning’ and ‘physical role’. These findings imply patients are able to perform all types of physical functioning more vigorously with less health-related limitations [15]. In MV disease patients, this is most likely related to a reduction in mitral regurgitation/mitral stenosis-associated dyspnoea [14]. Of note, as bodily pain is usually not associated with MV disease, this does not seem to play an important role in the preoperative phase. Furthermore, 1 year after surgery, bodily pain is infrequent, confirming previous findings at 1 year in patients undergoing FS MV surgery [16]. However, differences in pain outcomes between both approaches could potentially be present in the shorter-term [17], but the current study design did not allow for shorter-term QoL evaluation.

Similarly statistically significant—though less pronounced—differences were found for an increase in mental QoL at 1 year, overall. In the current registry, both FS and MIMVS were associated with an increase in mental QoL at 1 year. Although patients did improve in terms of mental health, this was less notable for ‘social functioning’ and the ‘emotional role’, implying some residual difficulties with work and daily activities as a result of emotional problems [15]. However, the important improvements in mental QoL are in line with previous studies, demonstrating a significant reduction in anxiety and posttraumatic stress after undergoing MV surgery, compared to conservatively treated patients [18]. Of note, in another prior study, lower ‘mental’ QoL was associated with an increased dissatisfaction regarding the sternotomy scar [17], but this did not seem important in the current registry.

In order to evaluate which patients might benefit less—in terms of QoL—from either surgical approach, regression analyses were performed for a loss of QoL. New-onset arrhythmia and baseline QoL were associated with a significant decrease in QoL (≥5 points). Indeed, especially postoperative AF, which is common after cardiac surgery in general and MV surgery in particular [19], is reported to have an important influence on QoL [20]. In the currently studied patient population, new onset of arrhythmia occurred more frequently in patients undergoing the FS approach. Furthermore, higher baseline SF scores were associated with a 1-year reduction in QoL. Although this might seem confusing, this paradoxical finding can more easily be explained in MV surgery. In the current era, MV surgery is even indicated in asymptomatic patients when mitral regurgitation is severe, and a durable repair is likely [21]. Consequently, this important patient group generally does not experience any impairment in daily life pre-operatively, but might be subjected to a relative reduction in longer-term QoL after surgery, in a trade-off to a restored life expectancy. Although these findings are only hypothesis-generating, from a mental perspective, such asymptomatic individuals might not regard themselves as a ‘patient’ prior to surgery, while the surgery transformed them into life-long cardiovascular patients requiring extensive follow-up and medical treatment.

Given the increasing importance of the patient’s point of view, QoL assessment should be considered in all studies evaluating cardiac surgical procedures. Indeed, the authors of the UK Mini trial should be applauded for their extensive efforts to study these outcomes in a randomized study setting [4]. Although the SF-scoring surveys are widely available, other—more MV disease-specific—questionnaires exist, evaluating heart failure-related QoL, such as the Kansas City Cardiomyopathy Questionnaire (KCCQ), more closely related to functional capacity and heart failure symptoms [22]. Still, such valve disease-specific questionnaires might be difficult to implement on a national level, as they are less likely to result in a homogeneous nationwide QoL assessment of cardiac surgical procedures. Finally, especially on a study-level, more objective measures of physical QoL and activity could be considered, such as the use of pre- and postoperative accelerometers or smartwatches [23, 24].

### Limitations

Unfortunately, QoL assessment was non-mandatory to register in the NHR. Therefore, only a selected number of patients (19.4% of eligible patients) completed both pre- and postoperative SF-forms, subjecting the results to potential selection bias. This potential bias extents to the exclusive inclusion of (i) responders and (ii) 1-year survivors, as only these patients completed both pre- and postoperative QoL assessment (survivorship bias). A comparison between responders and non-responders revealed an elevated operative risk in non-responders, driven by an increased rate of female sex, concomitant surgery and a decreased left ventricular function. In addition, only mandatory baseline parameters were completely reported. Therefore, potentially relevant data such as symptomatology or preoperative presence of AF were not recorded. Also, this study originated from a non-randomized registry, and allocation bias may play a role. Therefore, we did not perform statistical test for comparisons between FS and MIMVS as treatment groups for the outcome of QoL. Furthermore, as this registry ensures the anonymization of data, patients were not traceable to separate centres. Consequently, no comparisons could be made between centres.

The current registry did not allow for incorporation of shorter-term (i.e. 1-, 3- and/or 6-month) QoL outcomes. Hypothetically, shorter-term differences could be present between the surgical approaches, as was demonstrated by the UK Mini trial [4, 17], although conflicting evidence exists in related cardiac surgical procedures [25]. Furthermore, echocardiographic and additional clinical follow-up is not incorporated in this registry. As such, we could not evaluate improvement of heart failure symptoms, left ventricular function or other factors such as satisfaction with cosmetic results. Finally, the results of the regression analyses should be interpreted with caution as AUC was suboptimal, especially for the MCS regression analysis, and CIs of the ORs were relatively wide.

## CONCLUSION

Mitral valve surgery is associated with a significant improvement in mental and physical QoL, and baseline QoL and new onset of arrhythmia are associated with a clinically relevant reduction in QoL. Future research could focus on the standardized incorporation of QoL assessment in studies evaluating less-invasive or transcatheter mitral procedures, to evaluate their effectiveness from the patient’s perspective, in addition to objective clinical outcomes.

## Supplementary Material

ivae051_Supplementary_Data

## Data Availability

Data will be shared, upon reasonable request to the corresponding author, with the permission of the Netherlands Heart Registration. **Samuel Heuts:** Conception; Analysis; Interpretation; Writing original draft; Revision draft; Final approval to be published; Agreement to be accountable. **Jules R. Olsthoorn:** Conception; Acquisition; Analysis; Interpretation; Writing original draft; Revision draft; Final approval to be published; Agreement to be accountable. **Saskia Houterman:** Acquisition; Analysis; Interpretation; Supervision; Revision draft; Final approval to be published; Agreement to be accountable. **Maaike M. Roefs:** Acquisition; Analysis; Interpretation; Supervision; Revision draft; Final approval to be published; Agreement to be accountable. **Jos G. Maessen:** Conception; Acquisition; Supervision; Revision draft; Final approval to be published; Agreement to be accountable. **Peyman Sardari Nia:** Conception; Acquisition; Analysis; Interpretation; Supervision; Revision draft; Final approval to be published; Agreement to be accountable. Interactive CardioVascular and Thoracic Surgery thanks Tomislav Kopjar, Christian Schlensak, Roman Gottardi, Giuseppe Comentale and the other anonymous reviewers for their contribution to the peer review process of this article.

## ABBREVIATIONS

CI: Confidence interval
FS: Full sternotomy
KCCQ: Kansas City Cardiomyopathy Questionnaire
MIMVS: Minimally invasive mitral valve surgery
MV: Mitral valve
NHR: Netherlands Heart Registration
OR: Odds ratio
PROM: Patient-reported outcome measures
SF-12: 12-Item short form survey
SF-36: 36-item short form survey
QoL: Quality of life
