# Supplementary material for: One-year postprocedural quality of life following mitral valve surgery: data from The Netherlands Heart Registration
Source: Interdiscip Cardiovasc Thorac Surg. 2024 Mar 23;38(4):ivae051. doi: 10.1093/icvts/ivae051 (PMC11021809; doi:10.1093/icvts/ivae051)
Supplement: ivae051_Supplementary_Data [file ivae051_supplementary_data.docx]

SUPPLEMENTARY MATERIAL

***Content***

Supplementary Material 1 – Members of the NHR registration committee page 2

Supplementary Material 2 – Flowchart for patient inclusion page 3

Supplementary Material 3 – Differences between responders and non- page 4
 responders

Supplementary Material 4 – Sensitivity analysis for mitral valve repair (1) page 5

Supplementary Material 5 – Sensitivity analysis for mitral valve repair (2) page 6

Supplementary Material 6 – Sensitivity analysis for isolated MV surgery (1) page 7

Supplementary Material 7 – Sensitivity analysis for isolated MV surgery (2) page 8

Supplementary Material 1. Members of the Cardiothoracic Surgery Registration Committee of the Netherlands Heart Registration

| ^Dr. Bramer^ | ^Amphia Hospital Breda^ |
| --- | --- |
| ^Dr. van Boven^ | ^Amsterdam UMC, AMC^ |
| ^Dr. Vonk^ | ^Amsterdam UMC, VUmc^ |
| ^Dr. Koene^ | ^Catharina Hospital Eindhoven^ |
| ^Dr. Bekkers^ | ^Erasmus MC Rotterdam^ |
| ^Dr. Hoohenkerk^ | ^HagaZiekenhuis the Hague^ |
| ^Dr. Markou^ | ^Isala Zwolle^ |
| ^Dr. de Weger^ | ^Leids Universitair Medisch Centrum^ |
| ^Dr. Segers^ | ^Maastricht UMC+^ |
| ^Dr. Porta^ | ^Medisch Centrum Leeuwarden^ |
| ^Dr. Speekenbrink^ | ^Medisch Spectrum Twente^ |
| ^Dr. Stooker^ | ^OLVG, Amsterdam^ |
| ^Dr. Li^ | ^Radboud UMC Nijmegen^ |
| ^Dr. Daeter^ | ^St. Antonius Ziekenhuis Nieuwegein^ |
| ^Dr. van der Kaaij^ | ^UMC Utrecht^ |
| ^Dr. Douglas^ | ^Universitair Medisch Centrum Groningen^ |

Supplementary Material 2. Flowchart for patient inclusion.

*MIMVS: minimally invasive mitral valve surgery, NHR: Netherlands Heart Registration, QoL: quality of life.*

Supplementary Material 3. Differences between responders and non-responders in the Dutch registry.

|  | **Responders**  (n=485) | **Non-responders**  (n=2016) | p-value |
| --- | --- | --- | --- |
| ***Baseline*** |  |  |  |
| *Age (years)* | 66.0 [58.0-72.0] | 66.0 [57.0 – 73.0] | 0.87 |
| *Female sex (%)* | 190 (39.2) | 899 (44.6) | 0.03 |
| *BMI (kg/m^2^)* | 25.7 [23.2 – 28.0] | 25.3 [23.0 – 27.7] | 0.04 |
| *Diabetes Mellitus (%)* | 20 (4.1) | 157 (7.8) | 0.01 |
| *LVEF > 50% (%)* | 395 (81.4) | 1421 (70.5) | <0.001 |
| *COPD (%)* | 35 (7.2) | 185 (9.2) | 0.17 |
| *Peripheral arterial disease (%)* | 8 (1.6) | 55 (2.7) | 0.17 |
| *Endocarditis (%)* | 3 (0.6) | 29 (1.4) | 0.15 |
| *Recent myocardial infarction (<90 day, %)* | 2 (0.4) | 14 (0.7) | 0.48 |
| ***Procedure*** |  |  |  |
| *MVr (%)* | 391 (80.6) | 1579 (78.3) | 0.27 |
| *MVR (%)* | 94 (19.4) | 433 (21.5) | 0.31 |
| *Other procedure (%)* | 0 (0) | 1 (0.1) | 0.99 |
| *Unknown (%)* | 0 (0) | 3 (0.1) | 0.99 |
| *Rhythm surgery (%)* | 97 (20.0) | 376 (18.7) | 0.50 |
| *ASD closure (%)* | 15 (3.1) | 57 (2.8) | 0.75 |
| *TV surgery (%)* | 57 (11.8) | 518 (25.7) | <0.001 |
| ***Mortality risk*** |  |  |  |
| *EuroSCORE (log)* | 3.07 [2.08-5.38] | 3.47 [2.08 - 6.03] | 0.01 |

*ASD: atrial septal defect, BMI: body mass index, COPD: chronic obstructive pulmonary disease, EuroSCORE: European system for cardiac operative risk evaluation, iLVEF: left ventricular ejection fraction, MVr: mitral valve repair, MVR: mitral valve replacement, TV: tricuspid valve.*

Supplementary Material 4. Subgroup and sensitivity analysis for pre- and postoperative absolute QoL of patients undergoing mitral valve repair.

|  | **Preoperative** | **Postoperative** | **Increase/Decrease** | **p-value** |
| --- | --- | --- | --- | --- |
|  | **FS (n=226)** | | | |
| **PCS** | 58 [46-75] | 73 [59-88] | Increase | <0.001 |
| **MCS** | 67 [53-79] | 73 [62-90] | Increase | <0.001 |
|  | **MIMVS (n=165)** | | | |
| **PCS** | 59 [42-81] | 75 [63-88] | Increase | <0.001 |
| **MCS** | 63 [53-69] | 69 [59-81] | Increase | <0.001 |

*FS: full sternotomy, MCS: mental component score, MIMVS: minimally invasive mitral valve surgery, PCS: physical component score.*

Supplementary Material 5. Subgroup and sensitivity analysis for pre- and postoperative stratified QoL of patients undergoing mitral valve repair.

|  | **Total**  (n=391) | **FS** (n=226) | **MIMVS** (n=165) |
| --- | --- | --- | --- |
| **PCS**  *Decreased (%)*  *Unchanged (%)*  *Increased (%)* | 61 (16%)  80 (21%)  250 (64%) | 31 (14%)  48 (21%)  147 (65%) | 30 (18%)  32 (19%)  103 (62%) |
| **MCS**  *Decreased (%)*  *Unchanged (%)*  *Increased (%)* | 73 (19%)  105 (27%)  213 (55%) | 41 (18%)  65 (29%)  120 (53%) | 32 (19%)  40 (24%)  93 (56%) |

*FS: full sternotomy, MCS: mental component score, MIMVS: minimally invasive mitral valve surgery, PCS: physical component score.*

Supplementary Material 6. Subgroup and sensitivity analysis for pre- and postoperative absolute QoL of patients undergoing truly isolated mitral valve surgery.

|  | **Preoperative** | **Postoperative** | **Increase/Decrease** | **p-value** |
| --- | --- | --- | --- | --- |
|  | **FS (n=169)** | | | |
| **PCS** | 60 [46-78] | 72 [63-89] | Increase | <0.001 |
| **MCS** | 68 [53-79] | 74 [61-89] | Increase | <0.001 |
|  | **MIMVS (n=184)** | | | |
| **PCS** | 59 [51-69] | 66 [56-75] | Increase | <0.001 |
| **MCS** | 56 [38-75] | 75 [56-88] | Increase | <0.001 |

*FS: full sternotomy, MCS: mental component score, MIMVS: minimally invasive mitral valve surgery, PCS: physical component score.*

Supplementary Material 7. Subgroup and sensitivity analysis for pre- and postoperative stratified QoL of patients undergoing truly isolated mitral valve surgery

|  | **Total**  (n=353) | **FS** (n=169) | **MIMVS** (n=184) |
| --- | --- | --- | --- |
| **PCS**  *Decreased (%)*  *Unchanged (%)*  *Increased (%)* | 71 (20%)  97 (28%)  185 (52%) | 32 (19%)  48 (28%)  89 (53%) | 39 (21%)  49 (27%)  96 (52%) |
| **MCS**  *Decreased (%)*  *Unchanged (%)*  *Increased (%)* | 55 (16%)  68 (19%)  230 (65%) | 24 (14%)  35 (21%)  110 (65%) | 31 (17%)  33 (18%)  120 (65%) |

*FS: full sternotomy, MCS: mental component score, MIMVS: minimally invasive mitral valve surgery, PCS: physical component score.*
